# 2112. *In vitro* Activity of Cefiderocol and Comparator Agents Against Enterobacterales From United States Hospitals Stratified by Infection Type (2020-2022)

**DOI:** 10.1093/ofid/ofad500.1735

**Published:** 2023-11-27

**Authors:** Jason J Bryowsky, Boudewijn L DeJonge, Sean T Nguyen, Joshua Maher, Rodrigo E Mendes, Miki Takemura, Yoshinori Yamano

**Affiliations:** Shionogi Inc., Florham Park, New Jersey; Shionogi Inc., Florham Park, New Jersey; Shionogi Inc., Florham Park, New Jersey; JMI Laboratories, North Liberty, Iowa; JMI Laboratories, North Liberty, Iowa; Shionogi & Co., Ltd, Toyonaka, Osaka, Japan; Shionogi & Co., Ltd., Toyonaka, Osaka, Japan

## Abstract

**Background:**

Cefiderocol (CFDC) is a siderophore-conjugated cephalosporin with broad activity against Gram-negative bacteria including multi-drug resistant organisms. The *in vitro* activity of CFDC and comparator agents was evaluated against Enterobacterales isolates collected during 2020-2022 in the SENTRY Antimicrobial Surveillance Program, stratified by infection type.

**Figure:**
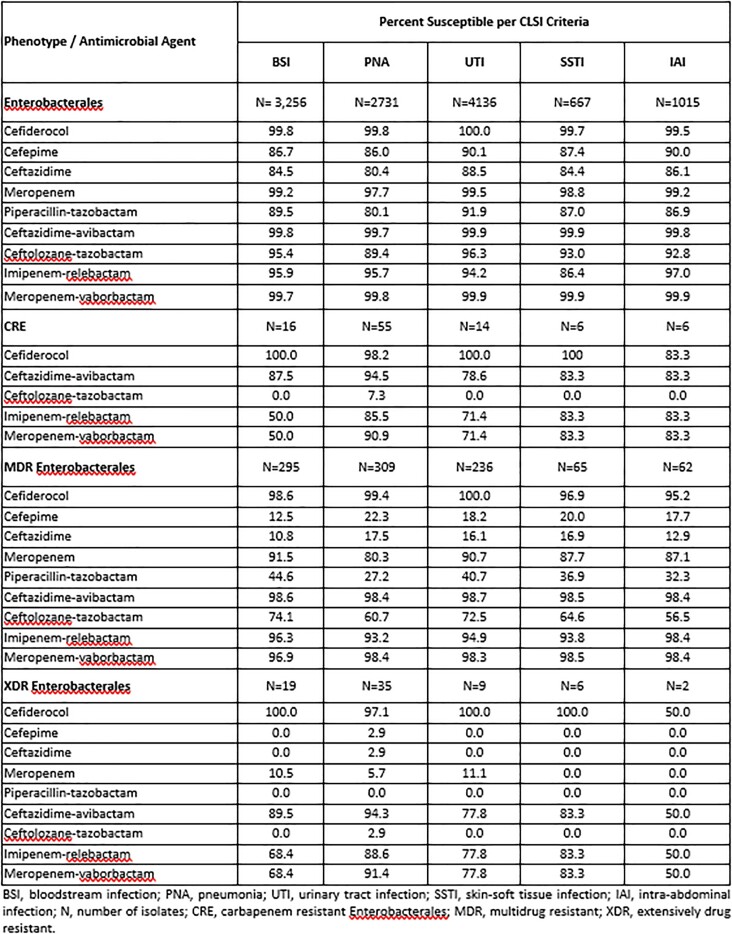

**Methods:**

11,805 Enterobacterales from the USA were tested for susceptibility (%S) using CLSI broth microdilution with cation-adjusted Mueller-Hinton broth (CAMHB) or iron-depleted CAMHB for CFDC. Comparator agents included β-lactam/β-lactamase inhibitor (BL/BLI) combinations ceftazidime-avibactam (CZA), ceftolozane-tazobactam (C/T), imipenem-relebactam (I-R) meropenem-vaborbactam (MVB), piperacillin-tazobactam (P/T) as well as meropenem (MEM), cefepime (FEP) and ceftazidime (CAZ). Susceptibility was interpreted according to 2022 CLSI & FDA breakpoints. Carbapenem resistant Enterobacterales (CRE) was defined as resistant to imipenem (IPM) and MEM. Multidrug-resistant (MDR) Enterobacterales was defined as nonsusceptible to at least 1 drug from ≥ 3 classes and extensively drug resistant (XDR) as susceptible to ≤ 2 classes per 2022 CLSI criteria.

**Results:**

The most common infection type from which isolates were collected was urinary tract infection (UTI; n=4,136), followed by bloodstream infection (BSI; n=3,256), pneumonia (PNA; n=2,731), intra-abdominal infections (IAI; n=1,015), and skin-soft tissue infections (SSTI; n=667). Among CRE and MDR isolates, CFDC was the most active agent ( > 98%S) for isolates from BSI, PNA, and UTI. Among XDR isolates from BSI and PNA, %S to CFDC ( > 97%) was highest amongst all agents tested. Of the Bl/BLIs, C/T and P/T demonstrated the lowest %S against CRE and MDR isolates from BSI, PNA, UTI and among XDR isolates from BSI and PNA.

**Conclusion:**

CFDC demonstrated high *in vitro* activity against Enterobacterales regardless of infection type. Susceptibility for comparator agents was generally lower against isolates with CRE, MDR, and XDR phenotypes. CFDC represents a potential early treatment option for infections caused by Enterobacterales with presumed or defined CRE, MDR, XDR phenotypes, regardless of infection type.

**Disclosures:**

**Jason J. Bryowsky, PharmD, MS**, Shionogi Inc.: Employee **Boudewijn L. DeJonge, PhD**, Shionogi Inc.: Employee **Sean T. Nguyen, PharmD**, Shionogi: Employee|Shionogi, Inc: Employee **Joshua Maher, PhD**, AbbVie: Grant/Research Support|Affinity Biosensors: Grant/Research Support|AimMax Therapeutics, Inc: Grant/Research Support|Alterity Therapeutics: Grant/Research Support|Amicrobe, Inc: Grant/Research Support|Arietis Pharma: Grant/Research Support|Armata Pharmaceuticals, Inc: Grant/Research Support|Astrellas Pharma, Inc.: Grant/Research Support|Basilea Pharmaceutica AG: Grant/Research Support|Becton Dickinson And Company: Grant/Research Support|bioMerieux, Inc: Grant/Research Support|Boost Biomes: Grant/Research Support|Diamond V: Grant/Research Support|Fedora Pharmaceuticals, Inc: Grant/Research Support|Iterum Therapeutics plc: Grant/Research Support|Johnson & Johnson: Grant/Research Support|Kaleido Biosciences, Inc.: Grant/Research Support|Meiji Seika Pharma Co. Ltd.: Grant/Research Support|National Institutes of Health: Grant/Research Support|Pfizer Inc.: Grant/Research Support|Roche Holding AG: Grant/Research Support|Shionogi Inc.: Grant/Research Support|Summmit Therapeutics, Inc.: Grant/Research Support|Zoetis Inc: Grant/Research Support **Rodrigo E. Mendes, PhD**, AbbVie: Grant/Research Support|Basilea: Grant/Research Support|Cipla: Grant/Research Support|Entasis: Grant/Research Support|GSK: Grant/Research Support|Paratek: Grant/Research Support|Pfizer: Grant/Research Support|Shionogi: Grant/Research Support **Miki Takemura, n/a**, Shionogi & Co., Ltd.: Stocks/Bonds **Yoshinori Yamano, PhD**, Shionogi HQ: Employee

